# Phylogenetic Findings Suggest Possible New Habitat and Routes of Infection of Human Eumyctoma

**DOI:** 10.1371/journal.pntd.0002229

**Published:** 2013-05-16

**Authors:** G. Sybren de Hoog, Sarah A. Ahmed, Mohammad J. Najafzadeh, Deanna A. Sutton, Maryam Saradeghi Keisari, Ahmed H. Fahal, Ursala Eberhardt, Gerard J. Verkleij, Lian Xin, Benjamin Stielow, Wendy W. J. van de Sande

**Affiliations:** 1 CBS Centraalbureau voor Schimmelcultures, KNAW Fungal Biodiversity Centre, Utrecht, The Netherlands; 2 Institute for Biodiversity and Ecosystem Dynamics, University of Amsterdam, Amsterdam, The Netherlands; 3 Department of Dermatology, the Second Affiliated Hospital, Sun Yat-Sen University, Guangzhou, Guangdong, People‚s Republic of China; 4 Peking University Health Science Center, Research Center for Medical Mycology, Beijing, People‚s Republic of China; 5 Department of Medical Microbiology, Faculty of Medical Laboratory Sciences, University of Khartoum, Khartoum, Sudan; 6 Department of Parasitology and Mycology, and Cancer Molecular Pathology Research Center, Ghaem Hospital, School of Medicine, Mashhad University of Medical Sciences, Mashhad, Iran; 7 Fungus Testing Laboratory, Department of Pathology, University of Texas Health Science Center at San Antonio, San Antonio, Texas, United States of America; 8 Mycetoma Research Centre, University of Khartoum, Khartoum, Sudan; 9 Department of Dermatology and Venereology, Union Hospital, Tongji Medical College, Huazhong Science and Technology University, Wuhan, People‚s Republic of China; 10 Erasmus MC, Department of Medical Microbiology and Infectious Diseases, Rotterdam, Netherlands; Fundação Oswaldo Cruz, Brazil

## Abstract

Eumycetoma is a traumatic fungal infection in tropical and subtropical areas that may lead to severe disability. *Madurella mycetomatis* is one of the prevalent etiologic agents in arid Northeastern Africa. The source of infection has not been clarified. Subcutaneous inoculation from plant thorns has been hypothesized, but attempts to detect the fungus in relevant material have remained unsuccessful. The present study aims to find clues to reveal the natural habitat of *Madurella* species using a phylogenetic approach, i.e. by comparison of neighboring taxa with known ecology. Four species of *Madurella* were included in a large data set of species of *Chaetomium*, *Chaetomidium*, *Thielavia*, and *Papulaspora* (n = 128) using sequences of the universal fungal barcode gene rDNA ITS and the partial LSU gene sequence. Our study demonstrates that *Madurella* species are nested within the *Chaetomiaceae*, a family of fungi that mainly inhabit animal dung, enriched soil, and indoor environments. We hypothesize that cattle dung, ubiquitously present in rural East Africa, plays a significant role in the ecology of *Madurella*. If cow dung is an essential factor in inoculation by *Madurella*, preventative measures may involve the use of appropriate footwear in addition to restructuring of villages to reduce the frequency of contact with etiologic agents of mycetoma. On the other hand, the *Chaetomiaceae* possess a hidden clinical potential which needs to be explored.

## Author Summary

Eumycetoma caused by *Madurella mycetomatis* is a common subcutaneous, mutilating fungal infection endemic in arid climate zones. Still there are many controversies on the route of infection, but traumatic inoculation of the subcutaneous tissue with the thorn or soil causative organism through minor skin trauma is a popular theory. This is due to the fact that, the origin and natural habitat of *Madurella* species, the prevalent mycetoma agents are still unknown. In order to predict the natural habitat of *M. mycetomatis* we investigated its phylogenetic relationships to species with known ecology. Two genes phylogeny based on LSU and ITS was performed for the species of the genus *Madurella* and representative genera from the family of *Chaetomiaceae*. Our findings confirmed that *Madurella* species are phylogenetically member of the family *Chaetomiaceae*. Members of this family are often found in dung and manure-enriched soil. We therefore suggest that animal dung, abundantly present in endemic villages, could be a possible niche for *Madurella* and plays an essential role in the onset of eumycetoma. This will help in understanding the origin of the disease and could be a base for future in depth study to investigate the presence of *Madurella* in dung from endemic areas.

## Introduction

**Figure 1:**
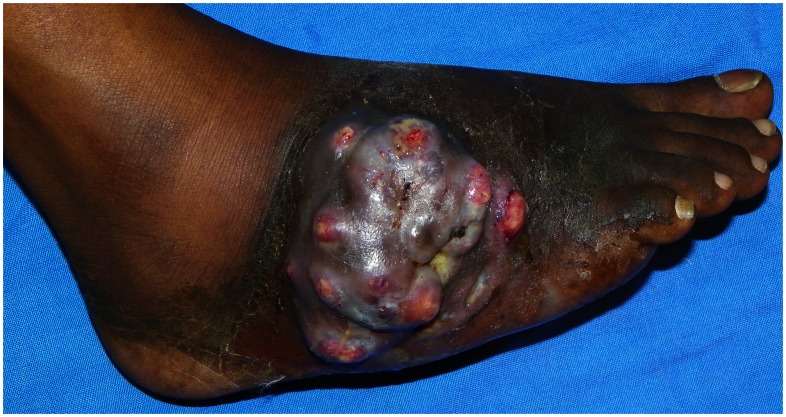
Eumycetoma showing granulomatous tumefaction of lateral aspect of right foot with sinus oozing black granules.

Eumycetoma is a subcutaneous disease with a high morbidity. It is prevalent in tropical and subtropical arid climate zones, with a focus in Northeastern Africa and particularly the Sudan [1]. Patients who develop advanced mycetoma of the extremities frequently become invalids due to the immobilizing nature of the disease (Fig. 1) [2]. Due to lack of social programs and poverty, patients become perpetually dependent on their family. Mycetoma can be caused by a variety of both bacteria (actinomycetoma) and fungi (eumycetoma) and is chronically progressive [1], [2]. eumycetoma is difficult to treat by chemotherapy, surgery frequently leads to mutilation, and relapse is common postoperatively. In the Sudan alone, 25% of the eumycetoma patients underwent amputation of the infected limb because of failure of therapy [3].

In order to reduce the morbidity of this disease, not only is an improvement in chemotherapy required, but also in the preventive measures. These might involve an efficient vaccine, as well as a reduction of contact with the causative agent. Gaining insight in the natural habitat of the prevalent Sudanese agent of mycetoma, *Madurella mycetomatis*, may lead to strategies to prevent introduction of causative agents into the skin and should reduce the burden of this disease in the endemic communities. However, the natural habitat of the prevalent Sudanese agent of mycetoma, *Madurella mycetomatis*, is unknown. The classical hypothesis is that aetiologic agents are traumatically introduced via thorn-pricks or with soil particles contaminated by the aetiologic agent, but *M. mycetomatis* has never been cultured from either thorns or soil. *Madurella* DNA was demonstrated in 17 out of 74 soil samples, and only in one out of 22 thorns tested [4]. Thus, the thorn-prick hypothesis seems less likely.

*Madurella mycetomatis* is thus far only known as sterile, melanized mycelium isolated from symptomatic patients. Isolates from subcutaneous infections that consist of dark hyphae are therefore routinely referred to as *‘Madurella’*, while those forming compact clumps of cells are traditionally identified as ‘*Papulaspora*’. Still no form of propagation, either sexual or clonal, is known for these fungi, except for some occasional, undiagnostic phialide-like cells [5]. There are many more causative agents of subcutaneous disorders which lack identifiable sporulation in culture. Today, identification options of such poorly structured fungi have increased with the development of molecular diagnostics. It has become clear that non-sporulating fungi are phylogenetically quite diverse. The melanized species causing black-grain mycetoma worldwide belong to at least two different orders of ascomycetes: the *Sordariales* and the *Pleosporales* [6].

In the present study we apply morphology-independent techniques to classify sterile agents of mycetoma in a phylogenetic scaffold of the fungi. This should lead to a better understanding of their ecology and pathology. Non-sporulating clinical isolates, provisionally deposited in two reference laboratories under the generic names *Madurella* and *Papulaspora*, were analyzed using the universal fungal barcode gene rDNA partial large subunit (LSU) and the internal transcribed spacer (ITS) regions. Since *Madurella mycetomatis* is a member of the order *Sordariales*, *Madurella pseudomycetomatis*, *M. fahalii* and *M. tropicana* most likely belong to the same order [7]. Phylogenies based on the mitochondrial genome confirmed the relationship to the *Sordariales*. Shared synteny was observed of genes and tRNAs in the mitochondrial genomes of *M. mycetomatis* and *Chaetomium thermophilum* [8]. *Chaetomium* is a large genus of *Sordariales* with more than 100 described species [9], but only very few species have been sequenced yet. In the present study we sequenced reference and additional clinical isolates of *Chaetomium* (ITS and LSU). Further members of the family *Chaetomiaceae* (*Sordariales*), including representatives of the genera *Achaetomium*, *Aporothielavia*, *Chaetomidium*, and *Thielavia* were selected to build up a framework of neighboring species to *Madurella*. Notably nearly all these fungi are ascosporulating only, producing elaborate fruiting bodies which cannot be expressed in human host tissue. Loss of the fruiting body thus immediately leads to sterile, *Madurella*-like cultures, rather than to a conidial counterpart as is the case in the majority of filamentous fungi. Comparison of ecological habitats of *Chaetomiaceae* was done in order to predict aspects of possible sources and routes of transmission of *Madurella* species.

## Materials and Methods

### Strains analysed

The analysis consists of 128 strains among which 60 strains of *Chaetomiaceae* contain presently available ex-type strains of described species deposited in the CBS culture collection. A total of 13 sterile filamentous isolates identified as *Madurella*, and one meristematic isolate, phenotypically identified as *Papulaspora* sp. were analyzed. The set was complemented with 54 clinical strains identified in this study (Supporting information; table S1). All clinical isolates included in our study were previously isolated from human sources and were taken from the CBS reference collection. Information on strains can be found at (www.cbs.knaw.nl)

### DNA extraction

About 10 mm^3^ fungal mass grown on agar surface were scraped in 2 ml screw cap vial containing 490 µl CTAB-buffer (2% CTAB, 100 mM Tris-HCL, 20 mM EDTA, 1.4 M NaCl) and 6–10 acid washed glass beads. In the subsequent step 10 µl of proteinase K (50 mg/ml) were added and the extraction buffer containing the sample vortexed for 2–5 minutes. The vials were incubated at 60°C for 60 minutes and vortexed again to ensure homogeneity of the sample. 500 µl of SEVAG (Chloroform∶Isoamylalcohol 24∶1) were added and the vials inverted repeatedly for at least two minutes. Vials were centrifuged at 14000 rpm (Eppendorf 5417R, Hamburg, Germany) for 10 minutes and the supernatant collected in new sterile vials with 0.55 volumes of ice cold 2-propanol and inverted several times. The precipitated total nucleic acids were centrifuged at 14000 rpm for 10 minutes. Finally, the pellets were washed with 70% ethanol, air- dried and re-suspended in 100 µl TE buffer.

### PCR and sequencing

The internal transcribed spacer (ITS) was amplified using the primers V9G and LS266 [10]. The resulting amplicons were bidirectionally sequenced with primers ITS1 and ITS4 [11]. The partial large ribosomal subunit (28S) was amplified with primer LR0R and LR5 and sequenced with the same primers [12]. A life Technologies Corp. 3730XL Sanger laboratory capillary electrophoresis system was used to retrieve the sequence data.

### Alignment and phylogenetic analysis

Trace files retrieved from bidirectional sequencing, were assembled and manually edited using Lasergene Seqman (DNASTAR, USA). A selection of 89 strains from the total data set was used for inferring the phylogenetic tree. Sequences were aligned with MUSCLE using the EMBL-EBI web server (http://www.ebi.ac.uk/Tools/msa/muscle/). A concatenated alignment was assembled for complete ITS (ITS1-5.8S-ITS2) and partial LSU sequences.

Bayesian and maximum likelihood analysis were performed with MrBayes v. 3.1.2 [13], and RAxML 7.2.8 respectively [14], [15]. MrBayes was run for 1 000 000 generations; one tree was saved per 100 of generations and burn-in was set for 25% of the saved trees. The 50% majority consensus tree was calculated and the final tree was edited using MEGA v. 5.05 [16]. Maximum likelihood was conducted using the CIPRES website (www.phylo.org), and GTR (General Time Reversible) model of nucleotide substitution was used; it is the only nucleotide substitution model in the RAxML software.

## Results

### Phylogenetic analysis

The analyzed data set comprised representative strains of the *Chaetomiaceae* [*Sordariales*] of both clinical and environmental origins (Supporting information; table S1).

**Figure 2:**
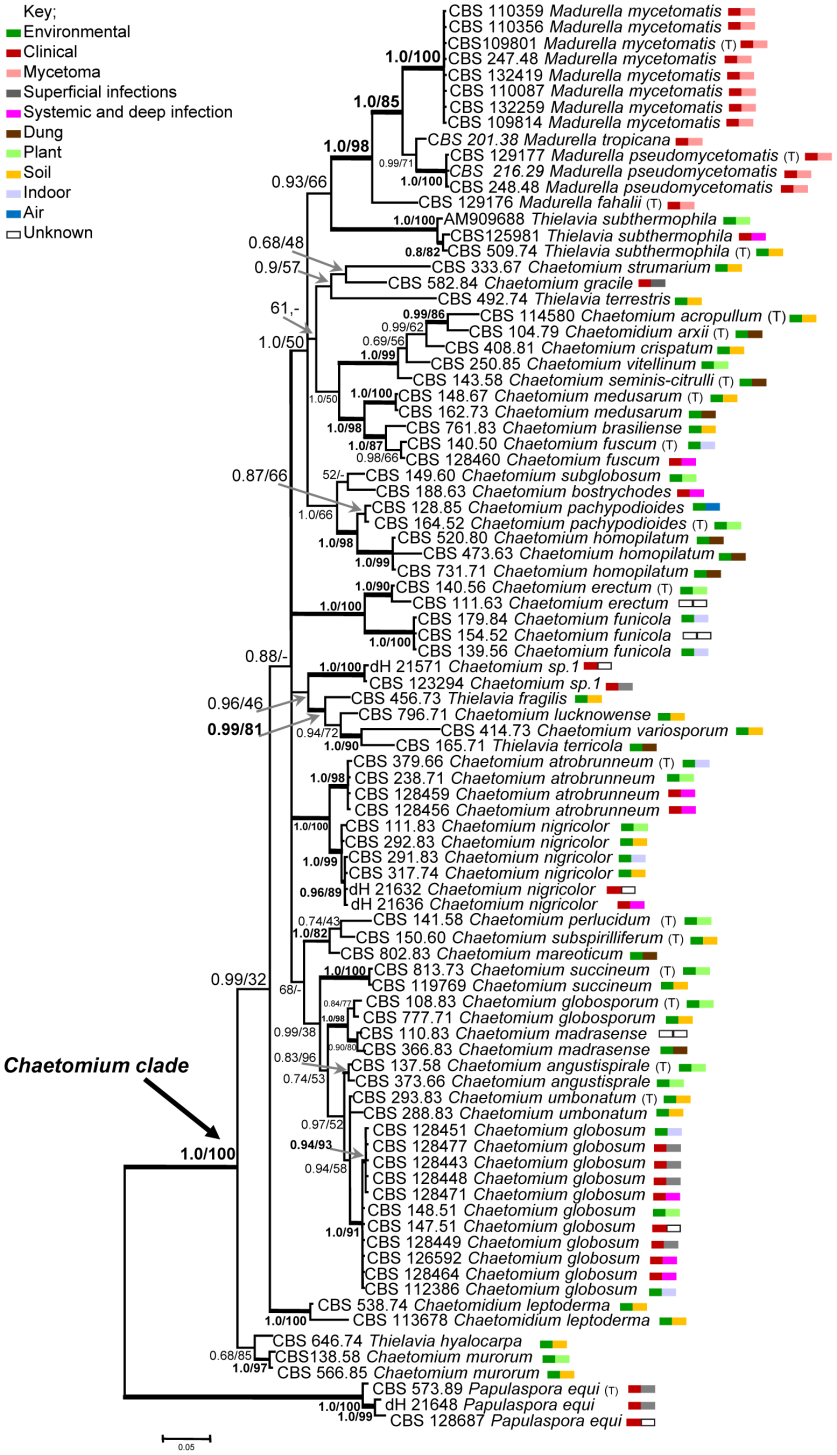
Phylogenetic tree resulting from Bayesian and maximum likelihood analysis of the combined ITS/LSU data set. Branches supported [>0.8 Bayesian probability, >80% Maximum likelihood] are drawn in bold. *Papulaspora equi* strains [CBS 573.89, CBS 128687, dH 21648] were used to root the tree. Type strain representing correct taxonomy [T].

Alignment of the combined genes sequences (ITS, LSU) consisted of 1,356 total characters in which 1029 were constant and 307 were variable. In our two-gene phylogeny most basal and internal branches show high Bayesian inference posterior probability values (BII PP) and maximum-likelihood bootstrap support (ML BS) respectively (Fig. 2). However, some internal branches of the *Chaetomiaceae* ingroup tree (split 0.88/-) comprising several clusters, e.g. for *C. atrobrunneum* and *C. nigricolor* (1.0/100), *Chaetomium* “sp. 1”, *C. lucknowense*, *C. variosporum*, *Thielavia terricola* and *T. fragilis* (0.96/46) as well as *C. errectum* and *C. funicola* (1.0/100), could not be fully resolved into dichotomies. The ingroup tree comprised a monophyletic cluster with four *Madurella* species with 1.0, 85% BII PP and ML BS, respectively, basal to *Thielavia subthermophila* (0.93/66). *Madurella* clustered within a large clade containing mostly environmental *Chaetomium* species which were distant from the type species of *Chaetomium* (*C. globosum*; Fig. 2). *Madurella fahalii* was identified as the closest taxon to the *Chaetomiaceae* at 6.0% ITS divergence from *Chaetomium nigricolor*. *Papulaspora equi*, known from three clinical isolates and identified by it is ex-type strain, was resolved basal to the grade comprising the *Chaetomium/Chaetomidium/Thielavia/Madurella* clades.

### Sequence identity and classification

The data set contained 38 ex-type and authentic strains. Twenty-two of these were usable to define each as OTU's (Operational taxonomical unit), while 16 were found to be identical to other described species defined by an ex-type isolate. Seven species, as delimited by sequence data, comprised more than one ex-type strain having identical sequences, rendering these species as provisional synonyms. Groups of isolates identified as the classical species *Chaetomium globosum*, described in the 19^th^ century without deposition of live material, did not contain an ex-type strain. In total, 29 *Chaetomium* species were judged to be distinct at the LSU/ITS level (Fig. 2), each being separated by several point mutations. Eight strains originating from clinical resources did not show identity to any known *Chaetomium* species and were therefore reported as ‘unknown *Chaetomium* sp.’ Three clinical isolates described as ‘*Chaetomium* sp. 1’, which had provisionally been identified as ‘*Papulospora* sp.’ on the basis of phenotypic characters, were found within the *Chaetomium* grade (Fig. 2, Supporting information; table S1).

All *Achaetomium* species were found to be synonyms of known *Chaetomium* species including ex-type strains of *Achaetomium nepalense*, *A. thermophilum*, and *A. strumarium*.

### Strain origin

The origins of 128 strains analyzed are summarized in table S1 (supporting information). A large quantity (40.6%; n = 52), were of environmental origin; about 7.0% (n = 9) were derived from animal dung, mainly of herbivores such as antelopes, goats, elephants, hares and rodents, but also of carnivores such as foxes. A percentage of 16.4% (n = 21) originated from soil either mixed with dung or decayed plant material, or from rhizosphere; 10.9% (n = 14) were derived from putrid plant material. Several species (*C. globosum*, *C. atrobrunneum*) were repeatedly isolated from indoor environments such as mouldy rugs and mattresses.

A total of 54.7% (n = 70) of the overall analyzed strains were from clinical samples. Forty-five out of 112 *Chaetomiaceae* strains of *Chaetomium*, *Chaetomidium* and *Thielavia* were infection-related, of which 49 strains originated from humans and 5 were veterinary isolates. Five out of eight strains identified as *C. atrobrunneum* were obtained from deep localizations including sputum, bronchial lavages and brain. *Chaetomium globosum* was frequently isolated from clinical or veterinary sources (24 strains where information about the origin was available).

In general, the clinical isolates were predominately isolated from the respiratory tract (9.4%, n = 12), possibly as asymptomatic colonizers. A large number of strains (22.7%, n = 29) were isolated from superficial samples including skin, hair, nails and eyes. Five isolates (3.9%) were derived from brain of four humans and one horse, and five (3.9%) strains were recovered from blood and lymph nodes. Infections reported as being subcutaneous were exceptional (0.78%, n = 1 from a wound); none of these were associated with production of grains in tissue.

Within the *Chaetomium* grade, one unnamed ‘*Chaetomium* sp. 1’ and four *Madurella* species were exclusively from clinical origin. Strains of ‘*Chaetomium* sp. 1’ were mainly associated with eye infections. All 13 strains identified as *Madurella* were derived from rural patients with subcutaneous eumycetoma with grain production.

## Discussion

The genus *Madurella*, comprising the species *M. mycetomatis*, *M. pseudomycetomatis*, *M. fahalii* and *M. tropicana*, was found to cluster within the *Chaetomiaceae*. In contrast to *Madurella*, most species of this family are able to produce elaborate fruiting bodies with characteristically shaped setae and ascospores. The impressive morphology of the ascomata suggests that species should be easily distinguishable by microscopic morphology, using the available classical, richly illustrated monographs [9], [17]. However, judging from our phylogenetic data (Fig. 2), molecular taxonomy matches poorly with morphology. At the generic level, the distinction between *Chaetomium*, *Achaetomium*, *Chaetomidium* and *Thielavia* is ambiguous, since several species of these genera clustered amidst *Chaetomium* species. Sometimes several ex-type strains of described taxa were found to have identical ITS sequences, suggesting that names should be reduced to synonymy. It may be concluded that molecular classification of *Chaetomiaceae* is significantly different from conventional taxonomy and extensive revision is needed at generic as well as at species levels. The position of *Madurella* as a derived clade within the family is unambiguous, and unexpected.

**Figure 3:**
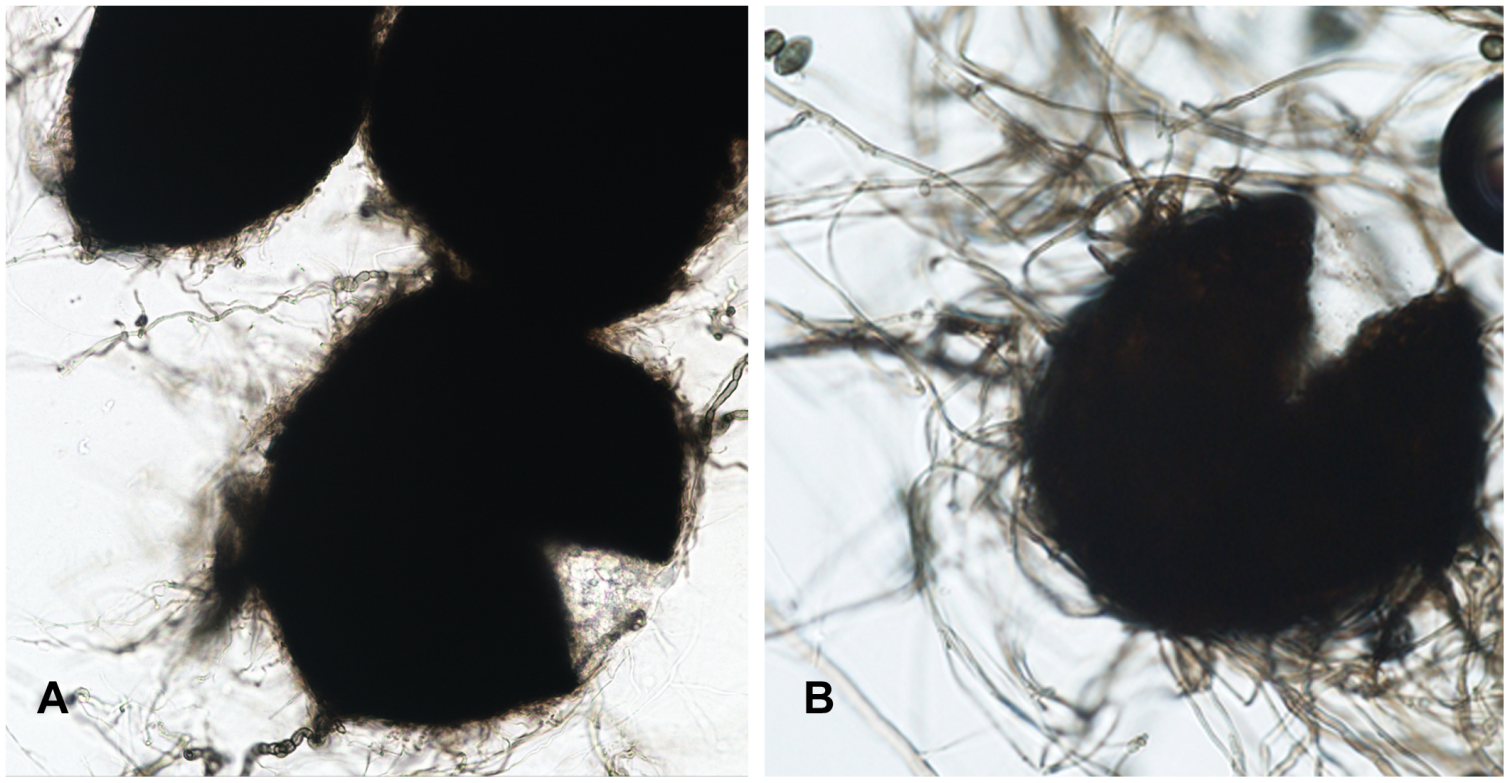
Structures produced by *Madurella mycetomatis* and *Thielavia subthermophila*. A: black sterile sclerotial bodies of *Madurella mycetomatis*. B: *Thielavia subthermophila* fruiting body (ascomata) from which ascospores are produced.

Most members of the *Chaetomiaceae* lack anamorph sporulation, or some scattered, undiagnostic phialides are present at most [9]. Thus, if strains lose the ability to produce their elaborate ascomata, they cannot be recognized as a *Chaetomium* species by morphological means, as in *Madurella*. Most of the clinical *Chaetomium* strains analyzed in the course of this study produced ascomata in culture, but some had remained sterile. The clinical strains of *Chaetomium* were responsible for cutaneous or systemic phaeohyphomycoses, but never produced eumycetoma. In contrast, strains of the *Madurella* subcluster, with four different molecular siblings, were consistently associated with eumycetoma. They were all sterile or produced some undiagnostic, phialide-like cells. Large structures resembling fruiting bodies were occasionally observed in *Madurella* (Fig. 3), but these did not have the ability to produce ascospores. The *Madurella* clade is morphologically not so far away from remaining *Chaetomiaceae*, and the position of *Madurella* within the *Chaetomiaceae* thus is explainable. The clade deviates however by producing grains in host subcutaneous tissue.

A consistent human pathogen is thus introduced in the family *Chaetomiaceae*. Traditionally, most species of the family were considered to be insignificant as agents of human disease. Of the ∼100 *Chaetomium* species described to date only five have repeatedly been associated with infection [5]. The majority of *Chaetomium* clinical strains analyzed in this study were probably transient colonizers or agents of mild superficial disorders. Twenty seven were involved in onychomycosis or cutaneous and eye infections in otherwise healthy individuals. This matches with literature data [18], [19]. In our data, *Chaetomium globosum* showed a definite bias towards superficial infection, with 17 out of 29 strains analyzed (supporting information; table S1). The species is able to degrade keratin by production of extracellular keratinases [20]. Fatal, disseminated and cerebral infections by *Chaetomiaceae* have also been reported. In the literature about 20 deep and disseminated cases were described, nearly all in immunocompromised and severely debilitated patients [21], [22]. Several *Chaetomium*-like fungi thus show rather pronounced pathology, sometimes with species-specific predilections.

Grain formation in tissue by *Chaetomiaceae* other than *Madurella* is not known. A single case of chromoblastomycosis by *Chaetomium funicola* was reported by Piepenbring et al. [23]. The few subcutaneous cases [24] all showed hyphae in tissue rather than the compact grains of *Madurella* eumycetoma. In contrast to *Madurella*, none of the infecting *Chaetomiaceae* was exclusively clinical; all contained environmental strains as well. If agents of black-grain mycetoma have a relatively limited distribution in the phylogeny of *Sordariales*, i.e. are clustered within a single family, *Chaetomiaceae*, one may hypothesize that these fungi are predisposed to human infection and thus are likely to share a set of fundamental virulence factors. Many members of *Chaetomiaceae* have their natural habitat in soil or on mammal dung. A possible explanation of their recurrent virulence may lie in physiological properties such as growth at the human body temperature of 37°C, and the production of secondary metabolites such as inhibitors of chemokines and TNF-α [25], [26]. Particularly the fatal brain infections, which were repeatedly reported in *Achaetomium strumarium* (synonym of *Chaetomium strumarium*) [27], [28], in *C. atrobrunneum* [19], and in *Thielavia subthermophila* [21], all belonging to the *Chaetomiaceae*, are remarkable. The hidden clinical diversity of the *Chaetomiaceae* urgently needs to be explored.

The role of mammal dung and dung-enriched soil is one of the prime ecological niches in the order *Sordariales*, and this also holds true for *Chaetomium* [29] (supporting information; table S1). Some species in the current study exclusively grow in dung, such as *Chaetomium homopilatum*. Multiple *Chaetomium* and *Thielavia* species have been isolated in East Africa from different kinds of dung, ranging from cow and horse to more exotic types of dung such as of elephant and wildebeest [30]. Conversely, the position of *Madurella* in *Chaetomiaceae* is informative for the natural habitat of this pathogen. In the highly endemic area in Sudan, *M. mycetomatis* has as yet not been cultured, whereas the isolation of some other causative agents of mycetoma, *Nocardia brasiliensis*, *Actinomadura madurae*, and *Streptomyces somaliensis* has been successful [31]. The causative agent of eumycetoma *Leptosphaeria senegalensis* has been recovered from thorns of *Acacia* species in West and Central Sub-Saharan Africa [32]. *Pseudallescheria boydii* has been recovered from polluted soil samples all over the world, including the endemic mycetoma regions [33], [34], [35]. For *Madurella mycetomatis* numerous isolation attempts from environmental sources were without success [4], [36]. Thirumalachar et al. [37] reported *M. mycetomatis* from soil in India, but the identification was based on scant phenotypic characters only. The difficulty in recovering *M. mycetomatis* from soil might indicate that pure soil is not the natural habitat for this fungus. Other possible habitats were thorny plant thorns, as plant material was occasionally found in human tissue [36], but this remains exceptional. Based on our study, association with cattle dung now seems to be an alternative option. *Madurella mycetomatis* apparently needs other culture media for isolation. Enrichment with dung might be a successful strategy. This hypothesis may be extended to *Madurella fahalii*, *M. tropicalis* and *M. pseudomycetomatis*, which are endemic in the arid climate zone of Northeastern Africa and are exclusively known from human mycetoma.

Providing insight into the taxonomic position and possible natural habitat of *Madurella* species changes our view regarding routes of infection and prevalent risk factors for human mycetoma. The Gezira region in the Sudan is highly endemic for eumycetoma by *M. mycetomatis* [1]. Most inhabitants live on cattle and camel husbandry and agriculture [38]. Local villages are characterized by an abundance of cattle, goats, sheep, dogs, chickens and donkeys [39]. Cows are raised mainly for their milk and are kept in pens surrounded by walls made of mud or thorny bushes. The floors of the pens are paved with dry feces, thorns and trash [39], and some human settlements are made of dried cow dung. The family house is usually in direct contact with the pen. Inhabitants of the villages mostly are barefoot among the thorny bushes. Traumatic introduction of coprophilic fungi via thorn pricks is thus a plausible scenario. Given the low frequency of *Madurella* on thorns, contamination of dung and its role as an adjuvant in inoculation seems likely. If cow dung is an essential factor in inoculation by *M. mycetomatis*, preventative measures may involve the use of appropriate footwear in addition to restructuring of villages by stricter separation of animal husbandry and human settlement to reduce the frequency of contact with mycetoma etiologic agents.

## Supporting Information

Table S1 **Name, reported type strains, source, origin, and GenBank accession numbers for the analysed strains.** dH: [G.S. de Hoog working collection] UTHSC [University of Texas Health Science Center]. All type strains marked with [T].
(DOCX)
