# Supplementary material for: Phylogenetic Findings Suggest Possible New Habitat and Routes of Infection of Human Eumyctoma
Source: PLoS Negl Trop Dis. 2013 May 16;7(5):e2229. doi: 10.1371/journal.pntd.0002229 (PMC3656121; doi:10.1371/journal.pntd.0002229)
Supplement: Table S1 — Name, reported type strains, source, origin, and GenBank accession numbers for the analysed strains. dH: [G.S. de Hoog working collection] UTHSC [University of Texas Health Science Center]. All type strains marked with [T]. (DOCX) [file pntd.0002229.s001.docx]

| **Isolate Name** | **Isolate No** | **Other collection number** | **Current and synonyms type species** | **Isolate Source** | **Isolate Region** | **ITS** | **LSU** |
| --- | --- | --- | --- | --- | --- | --- | --- |
| *Chaetomidium arxii* | CBS 104.79 [T] |  | Type of *Chaetomidium arxii* | Dung | USA | JX280770 | FJ666359 |
| *Chaetomidium leptoderma* | CBS 538.74 [T] |  | Type of *Thielavia leptoderma* | Soil | England | AF096171 | AF096186 |
| *Chaetomidium leptoderma* | CBS 113678 [T] |  | Type of *Chaetomidium galaicum* | Soil | Spain | JN573175 | FJ666361 |
| *Chaetomium acropullum* | CBS 114580 [T] |  | Type of *Chaetomium acropullum* | Soil | China | JX280763 | JX280662 |
| *Chaetomium angustispirale* | CBS 137.58 [T] |  | Type of *Chaetomium angustispirale* | Plant | Russia | JX280764 | JX280663 |
| *Chaetomium angustisprale* | CBS 373.66 [T] |  | Type of *Chaetomium spiculipilium* | Plant | USA | JX280765 | JX280664 |
| *Chaetomium angustispirale* | dH 21623 | UTHSC 07-1348 |  | Nail | USA | JX280767 |  |
| *Chaetomium angustispirale* | CBS 128486 | UTHSC 08-622 |  | Bronchial lavage | USA | JX280768 |  |
| *Chaetomium angustispirale* | CBS 128487 | UTHSC 07-3907 |  | Nail | USA | JX280769 |  |
| *Chaetomium angustispirale* | CBS 128465 | UTHSC 06-454 |  | Nail | USA | JX280766 | JX280665 |
| *Chaetomium atrobrunneum* | CBS 379.66 [T] |  | Type of *Chaetomium atrobrunneum* | Mouldy mattress | SolomonIslands | JX280771 | JX280666 |
| *Chaetomium atrobrunneum* | CBS 238.71 [T] |  | Type of *Chaetomium rectopilium* | Plant/mushroom | Switzerland | JX280772 | JX280667 |
| *Chaetomium atrobrunneum* | CBS 128475 | UTHSC 09-2474 |  | Sputum | United State | JX280775 | JX280670 |
| *Chaetomium atrobrunneum* | CBS 128474 | UTHSC 09-101 |  | Right lung | United State | JX280776 | JX280671 |
| *Chaetomium atrobrunneum* | CBS 128692 |  |  | Unknown | Spain | JX280778 | JX280672 |
| *Chaetomium atrobrunneum* | CBS 128481 | UTHSC 04-1606 |  | Bronchial lavage | United State | JX280777 |  |
| *Chaetomium atrobrunneum* | CBS 128459 | UTHSC 01-329 |  | Brain | USA | JX280773 | JX280668 |
| *Chaetomium atrobrunneum* | CBS 128456 | UTHSC 07-175 |  | Bronchial lavage | USA | JX280774 | JX280669 |
| *Chaetomium bostrychodes* | CBS 188.63 |  |  | Sputum |  | JX280779 | JX280673 |
| *Chaetomium brasiliense* | CBS 761.83 |  |  | Soil | Egypt | X280780 | JX280674 |
| *Chaetomium crispatum* | CBS 408.81 |  |  | Soil | Netherlands | JX280781 | JX280675 |
| *Chaetomium erectum* | CBS 140.56 [T] |  | Type of *Chaetomium erectum* | Plant | USA | HM449044 | HM449058 |
| *Chaetomium erectum* | CBS 111.63 |  |  | Unknown | Belgium | JX280782 | JX280676 |
| *Chaetomium funicola* | CBS 154.52 |  |  | Unknown |  | JX280783 | JX280677 |
| *Chaetomium funicola* | CBS 139.56 [T] |  | Type of *Chaetomium causiiforme* | Cloths | SolomonIslands | JX280784 | JX280678 |
| *Chaetomium funicola* | CBS 179.84 [T] |  | Type of *Chaetomium variostiolatum* | Tarpaulin | NewGuinea | JX280785 | JX280679 |
| *Chaetomium funicola* | CBS 128488 | UTHSC 03-2605 |  | Nose | United State | JX280786 |  |
| *Chaetomium fuscum* | CBS 140.50 [T] |  | Type of *Chaetomium fuscum* | Moist jute cloth | India | JX280787 | AF286396 |
| *Chaetomium fuscum* | CBS 128480 | UTHSC 04-987 |  | Knee | United State | JX280789 | JX280681 |
| *Chaetomium fuscum* | CBS 128460 | UTHSC 03-1854 |  | Pleural fluid | USA | JX280788 | JX280680 |
| *Chaetomium globosum* | CBS 126592 |  |  | Blood | India | JX280793 | JX280686 |
| *Chaetomium globosum* | CBS 128471 | UTHSC 05-1845 |  | Lymph node | USA | JX280801 | JX280687 |
| *Chaetomium globosum* | CBS 128451 | UTHSC 06-1791 |  | Contact lens | USA | JX280794 | JX280688 |
| *Chaetomium globosum* | CBS 128448 | UTHSC 09-405 |  | Scalp | USA | JX280800 | JX280689 |
| *Chaetomium globosum* | CBS 128464 | UTHSC 09-3118 |  | Mediastinum | USA | JX280796 | JX280691 |
| *Chaetomium globosum* | CBS 128477 | UTHSC 10-814 |  | Skin | USA | JX280797 | JX280692 |
| *Chaetomium globosum* | CBS 128443 | UTHSC 03-824 |  | Nail | USA | JX280798 | JX280693 |
| *Chaetomium globosum* | CBS 128449 | UTHSC 06-2072 |  | Nail | USA | JX280795 | JX280690 |
| *Chaetomium globosum* | CBS 112386 |  |  | Indoor environment | Germany | JX280792 | JX280685 |
| *Chaetomium globosum* | CBS 148.51 |  |  | Plant | USA | GU563374 | JX280684 |
| *Chaetomium globosum* | CBS 128454 | UTHSC 03-611 |  | Scalp | USA | JX280803 | JX280694 |
| *Chaetomium globosum* | CBS 128493 | UTHSC 03-1338 |  | Nail | USA | JX280804 |  |
| *Chaetomium globosum* | CBS 128453 | UTHSC 03-1750 |  | Scalp | USA | JX280805 | JX280695 |
| *Chaetomium globosum* | CBS 128450 | UTHSC 03-1914 |  | Alligator skin | USA | JX280806 | JX280696 |
| *Chaetomium globosum* | CBS 128470 | UTHSC 05-1459 |  | Nail | USA | JX280807 | JX280697 |
| *Chaetomium globosum* | CBS 128497 | UTHSC 07-689 |  | Nail | USA | JX280808 |  |
| *Chaetomium globosum* | CBS 128476 | UTHSC 07-720 |  | Blood | USA | JX280809 | JX280698 |
| *Chaetomium globosum* | CBS 128469 | UTHSC 07-1090 |  | Nail | USA | JX280810 | JX280699 |
| *Chaetomium globosum* | CBS 128447 | UTHSC 07-1026 |  | Nail | USA | JX280811 | JX280700 |
| *Chaetomium globosum* | CBS 128446 | UTHSC 07-2213 |  | Nail | USA | JX280812 | JX280701 |
| *Chaetomium globosum* | CBS 128452 | UTHSC 08-954 |  | Blood | USA | JX280813 | JX280702 |
| *Chaetomium globosum* | CBS 128463 | UTHSC 07-477 |  | Nail | USA | JX280814 | JX280703 |
| *Chaetomium globosum* | CBS 128693 |  |  | Unknown | Spain | JX280815 | JX280704 |
| *Chaetomium globosum* | CBS 128445 | UTHSC 03-1916 |  | Alligator skin | USA | JX280816 | JX280705 |
| *Chaetomium globosum* | CBS 128694 |  |  | Unknown | Spain | JX280817 |  |
| *Chaetomium globosum* | CBS 128479 | UTHSC 04-1189 |  | Nail | USA | JX280818 |  |
| *Chaetomium globosum* | CBS 128485 | UTHSC 06-669 |  | Sinus | USA | JX280802 |  |
| *Chaetomium globosum* | CBS 128444 | UTHSC 02-785 |  | Nail | USA | JX280819 | JX280706 |
| *Chaetomium globosum* | CBS 147.51 |  |  | man | Germany | JX280799 | JX280707 |
| *Chaetomium globosporum* | CBS 108.83 [T] |  | Type of *Chaetomium globosporum* | Plant | India | JX280820 | JX280708 |
| *Chaetomium globosporum* | CBS 777.71 |  |  | Soil | Israel | JX280822 | JX280709 |
| *Chaetomium globosporum* | CBS 128494 | UTHSC 10-726 |  | Cornea | USA | JX280821 |  |
| *Chaetomium gracile* | CBS 582.84 |  |  | Nail | Netherlands | JX280823 | JX280710 |
| *Chaetomium homopilatum* | CBS 731.71 |  |  | Dung | India | JX280825 | JX280712 |
| *Chaetomium homopilatum* | CBS 520.80 |  |  | Dung | India | JX280826 | JX280713 |
| *Chaetomium homopilatum* | CBS 473.63 [T] |  | Type of *Chaetomium biapiculatum* | Dung | India | JX280824 | JX280711 |
| *Chaetomium lucknowense* | CBS 796.71 |  |  | Soil | Egypt | JX280827 | JX280714 |
| *Chaetomium madrasense* | CBS 110.83 [T] |  | Type of *Chaetomium gibberosporum* | Unknown |  | JX280790 | JX280683 |
| *Chaetomium madrasense* | CBS 366.83 |  |  | Dung | France | JX280791 | JX280682 |
| *Chaetomium mareoticum* | CBS 802.83 |  |  | Dung | Israel | JX280828 | JX280715 |
| *Chaetomium medusarum* | CBS 148.67 [T] |  | Type of *Chaetomium medusarum* | Soil | Zaire | JX280829 | JX280716 |
| *Chaetomium medusarum* | CBS 162.73 |  |  | Dung | East Africa | JX280830 | JX280717 |
| *Chaetomium murorum* | CBS 566.85 |  |  | Soil | Kenya | JX280832 | JX280719 |
| *Chaetomium murorum* | CBS 138.58 |  |  | Plant | Mongolia | JX280831 | JX280718 |
| *Chaetomium nigricolor* | CBS 317.74 [T] |  | Type of *Chaetomium verrucichaeta* | Soil | India | JX280836 | JX280720 |
| *Chaetomium nigricolor* | CBS 111.83 [T] |  | Type of *Chaetomium pseudoerraticum* | Plant | India | JX280837 | JX280721 |
| *Chaetomium nigricolor* | CBS 128478 | UTHSC 08-2214 |  | Biopsy | USA | JX280834 | JX280722 |
| *Chaetomium nigricolor* | CBS 128455 | UTHSC 09-1153 |  | Trachaea | USA | JX280835 | JX280723 |
| *Chaetomium nigricolor* | CBS 291.83 [T] |  | Type of *Chaetomium amberpetense* | Paper | India | JX280838 | JX280724 |
| *Chaetomium nigricolor* | CBS 292.83 [T] |  | Type of *Chaetomium vitis* | Soil | India | JX280833 | JX280725 |
| *Chaetomium pachypodioides* | CBS 128.85 [T] |  | Type of *Chaetomium intricatum* | Air | British Columbia | JX280839 | JX280726 |
| *Chaetomium pachypodioides* | CBS 164.52 [T] |  | Type of *Chaetomium pachypodioides* | Plant | Tennessee | GQ922526 | JX280727 |
| *Chaetomium perlucidum* | CBS 141.58 [T] |  | Type of *Chaetomium perlucidum* | Plant | Ukraine | JX280840 | JX280728 |
| *Chaetomium seminis-citrulli* | CBS 143.58 [T] |  | Type of *Chaetomium semenis-citrulli* | Dung | Turkmenistan | JX280841 | JX280729 |
| *Chaetomium sp.1 /Papulaspora sp.* | dH 21571 |  |  | Clinical |  | JX280842 | JX280730 |
| *Chaetomium sp.1 /Papulaspora sp.* | CBS123294 |  |  | keratitis | New Mexico | HQ906667 | JX280731 |
| *Chaetomium sp.1 /Papulaspora sp.* | CBS 128466 | UTHSC 07-434 |  | Corneal ulcer |  | JX280843 | JX280732 |
| *Chaetomium sp.2* | CBS 128462 | UTHSC 03-1508 |  | Bronchial ulcer |  | JX280844 | JX280733 |
| *Chaetomium sp.3* | CBS 128461 | UTHSC 03-1917 |  | Alligator skin | USA | JX280845 | JX280734 |
| *Chaetomium sp.4* | CBS 128492 | UTHSC 07-3593 |  | Neck | USA | JX280846 |  |
| *Chaetomium sp.5* | dH 21637 | UTHSC 09-1614 |  | Wound | USA | JX280847 |  |
| *Chaetomium sp.6* | dH 21633 | UTHSC 08-3228 |  | Brain | USA | JX280848 |  |
| *Chaetomium sp.7* | CBS 128489 | UTHSC 03-1339 |  | Hair, mammal | USA | JX280849 |  |
| *Chaetomium sp.8* | CBS 128491 | UTHSC 07-3656 |  | Brain | USA | JX280850 |  |
| *Chaetomium strumarium* | CBS 333.67 [T] |  | Type of *Achaetomium strumarium* | soil | India | AY681204 | AY681170 |
| *Chaetomium strumarium* | dH 21642 | UTHSC 10-1100 |  | Brain, horse | USA | JX280851 |  |
| *Chaetomium subglobosum* | CBS 149.60 [T] |  | Type of *Chaetomium subglobosum* | Plant | Russia | JX280852 | HM751083 |
| *Chaetomium subspirilliferum* | CBS 150.60 [T] |  | Type of *Chaetomium subspirilliferum* | Soil | Russia | JX280853 | JX280735 |
| *Chaetomium succineum* | CBS 813.73[T] |  | Type of *Chaetomium succineum* | Plant | USA | JX280854 | JX280736 |
| *Chaetomium succineum* | CBS 119769 |  |  | Soil | China | JX280855 | JX280737 |
| *Chaetomium umbonatum* | CBS 293.83 [T] |  | Type of *Chaetomium umbonatum* | Soil | Canada | JX280856 | JX280738 |
| *Chaetomium umbonatum* | CBS 288.83 [T] |  | Type of *Achaetomium nepalense* | Soil | Nepal | JX280857 | JX280739 |
| *Chaetomium umbonatum* | CBS 128484 | UTHSC 08-1518 |  | Scalp | USA | JX280858 |  |
| *Chaetomium vitellinum* | CBS 250.85 [T] |  | Type of *Achaetomium thermophilum* | Plant | India | JX280859 | JX280740 |
| *Chaetomium variosporum* | CBS 414.73 [T] |  | Type of *Chaetomium variosporum* | Soil | India | JX280860 | JX280741 |
| *Madurella fahaliii* | CBS129176[T] |  | Type of *Madurella fahaliii* | Mycetoma | Sudan | JN573178 | JX280751 |
| *Madurella mycetomatis* | CBS 110359 |  |  | Mycetoma | Mali | JX280861 | JX280746 |
| *Madurella mycetomatis* | CBS 110356 |  |  | Mycetoma | Mali | JX280862 | JX280747 |
| *Madurella mycetomatis* | CBS 247.48 |  |  | mycetoma |  | JX280863 | JX280745 |
| *Madurella mycetomatis* | CBS 110087 |  |  | Mycetoma | Sudan | JX280865 | JX280742 |
| *Madurella mycetomatis* | CBS 109801 [T] |  | Type of *Madurella mycetomatis* | Mycetoma | Sudan | DQ836767 | JX280743 |
| *Madurella mycetomatis* | CBS 132419 |  |  | mycetoma | India | JX280864 | JX280748 |
| *Madurella mycetomatis* | CBS 132259 |  |  | Mycetoma | Sudan | JX280866 | JX280749 |
| *Madurella mycetomatis* | CBS 109814 |  |  | Mycetoma | Sudan | JX280867 | JX280744 |
| *Madurella pseudomycetomatis* | CBS 248.48 |  |  | Mycetoma | New Mexico | JX280868 | JX280753 |
| *Madurella pseudomycetomatis* | CBS 129177 [T] |  | Type of *Madurella mycetomatis* | Mycetoma | China | EU815933 | JX280752 |
| *Madurella pseudomycetomatis* | CBS 216.29 |  |  | Mycetoma |  | JN573187 | JX280754 |
| *Madurella tropicana* | CBS 201.38 |  |  | Mycetoma | Indonesia | JX280869 | JX280750 |
| *Papulaspora equi* | CBS 573.89[T] |  | Type of *Papulaspora equi* | Ocular lesion | USA | JX280870 | JX280755 |
| *Papulaspora equi* | CBS 128684 |  |  | Eye | USA | JX280871 | JX280756 |
| *Papulaspora equi* | CBS 128687 |  |  | Buttock |  | JX280872 | JX280757 |
| *Thielavia fragilis* | CBS 456.73[T] |  | Type of *Chaetomidium fragile* | Soil |  | AJ271578 | JX280758 |
| *Thielavia hyalocarpa* | CBS 646.74 |  |  | Soil | Egypt | AJ271583 | JX280759 |
| *Thielavia subthermophila* | CBS 125981 |  |  | Brain |  | HM448441 | HM448442 |
| *Thielavia subthermophila* | AM909688 |  |  | Plant |  | AM909688 | AM909688 |
| *Thielavia subthermophila* | CBS 509.74 [T] |  | Type of *Thielavia subthermophila* | Soil | Egypt | JX280873 | JX280760 |
| *Thielavia terrestris* | CBS 492.74 |  |  | Soil | Japan | JX280874 | JX280761 |
| *Thielavia terricola* | CBS 165.71 |  |  | Dung | Canada | JX280875 | JX280762 |
